# Effect of muscle strength on deep vein thrombosis: A Mendelian randomization study

**DOI:** 10.1097/MD.0000000000040138

**Published:** 2024-11-08

**Authors:** Yijia Gao, Hui Chen, Daoze Ke, Changfan Li, Ziwei Jiang, Bin Pu

**Affiliations:** a The First Affiliated Hospital, Guangzhou University of Chinese Medicine, Guangzhou, Guangdong Province, China; b Guangzhou University of Chinese Medicine, Guangzhou, Guangdong Province, China; c Department of Orthopedics, Suining Traditional Chinese Medicine Hospital Affiliated to North Sichuan Medical College, Suining, Sichuan Province, China.

**Keywords:** deep vein thrombosis, instrumental variables, Mendelian randomization, muscle strength

## Abstract

Deep vein thrombosis (DVT) is a serious condition characterized by blood clots in deep veins, posing a significant public health burden. Muscle strength has been implicated as a potential risk factor for DVT due to its influence on venous return. This study aims to investigate the causal association between muscle strength and DVT using a Mendelian randomization (MR) approach, leveraging genetic variants as instrumental variables (IVs). We conducted a 2-sample MR analysis using genome-wide association study (GWAS) data for hand-grip strength and DVT. IVs were selected based on their significant associations with muscle strength and DVT, as well as their linkage disequilibrium patterns. We employed statistical methods including inverse-variance weighting (IVW), MR-Egger, and weighted median to address pleiotropy bias. Sensitivity analyses were conducted to evaluate the robustness of the results. A total of 21 and 14 independent IVs were identified for hand grip strength (EWGSOP) and hand grip strength (FNIH), respectively. IVW analysis revealed a consistent causal and negative association between both definitions of hand grip strength and DVT (EWGSOP: OR = 0.702, 95% CI: 0.511–0.964, *P* = .029; FNIH: OR = 0.715, 95% CI: 0.570–0.898, *P* = .004). No directional pleiotropy was detected in MR-Egger and MR-PRESSO analyses for either definition (EWGSOP: MR-Egger Intercept *P* = .516; MR-PRESSO global test *P* = .162; FNIH: MR-Egger Intercept *P* = .569; MR-PRESSO global test *P* = .371).Sensitivity analyses demonstrated the stability of the causal effect estimates, with little influence from individual IVs. The MR analysis provided evidence of a causal association between muscle strength and DVT risk, suggesting that increasing muscle strength may have a protective effect. These findings have implications for preventive strategies and the promotion of resistance exercises and muscle-strengthening activities. Further research and validation of these results could inform clinical guidelines and interventions for DVT prevention.

## 1. Introduction

Deep vein thrombosis (DVT) is a prevalent and potentially life-threatening condition characterized by the formation of blood clots within deep veins, primarily in the lower extremities.^[1–3]^ With an estimated annual incidence of 1 to 2 cases per 1000 individuals, DVT poses a significant burden on public health.^[4,5]^ Timely identification and effective prevention of DVT are of paramount importance due to its associated morbidity, mortality, and potential for pulmonary embolism.^[6]^

Understanding the modifiable risk factors for DVT is crucial in developing targeted interventions and improving patient outcomes.^[7,8]^ Among the potential factors, muscle strength has emerged as a compelling candidate influencing DVT risk.^[9,10]^ Evidence indicates that reduced muscle mass in the thighs,^[11]^ weak calf pump function,^[12]^ and weak grip strength^[13]^ are associated with an increased risk of DVT. Additionally, resistance exercises have been suggested as measures to maximize mechanical benefits for early mobilization and DVT prophylaxis.^[14,15]^ The intricate relationship between muscle strength and DVT stems from the physiological role of muscles in promoting venous return and enhancing blood flow.^[16,17]^ Consequently, impaired muscle strength and diminished muscle function could compromise venous return, predisposing individuals to a heightened risk of DVT.

Mendelian randomization (MR) is a powerful analytical approach that leverages genetic variants as instrumental variables (IVs) to explore causal relationships between exposure and outcome variables.^[18]^ Genetic variants are randomly allocated at conception and are not influenced by confounding factors or reverse causation, making them ideal instruments to assess causal relationships.^[19]^ Applying MR to study the association between muscle strength and DVT can provide valuable insights by reducing biases commonly found in observational studies.

The aim of this study is to investigate the causal association between muscle strength and DVT using a MR approach. By leveraging genetic variants associated with muscle strength as IVs, we seek to assess whether increasing muscle strength has a causal impact on reducing DVT risk. The findings of this study may provide valuable insights into the preventive strategies for DVT and guide recommendations for resistance exercise and muscle-strengthening activities.

## 2. Method

### 2.1. Study design

**Figure 1.**
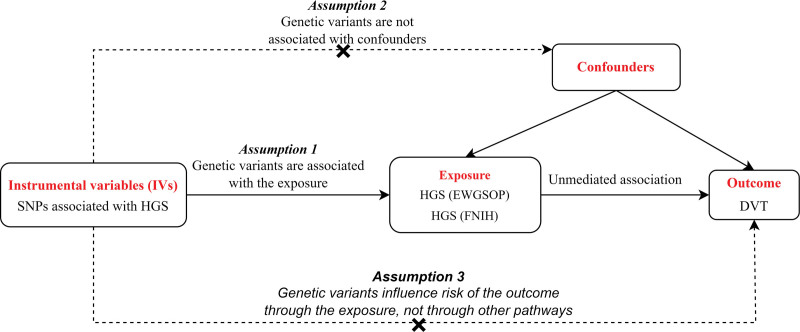
Diagram for key assumptions of MR analyses. DVT = deep vein thrombosis, HGS = hand grip strength, IVs = instrumental variables, MR = Mendelian randomization, SNPs = single nucleotide polymorphisms.

In our study, we made several assumptions to conduct the instrumental variable analysis. Assumption 1 was based on the strong correlation between the instrumental variable and the exposure factor and derivate the causal effects using IVs instead of exposure factors. Assumption 2 stated that the instrumental variable was not associated with any confounding factors affecting the relationship between the exposure and outcome. Assumption 3 suggested that the instrumental variable had no direct effect on the outcome unless it was mediated through its association with the exposure. Based on the 3 core assumptions, it is possible to ensure the logical correctness of the MR analysis and to avoid the influence of potential confounding factors on the results. We focused on muscle strength as the exposure factor. We identified single nucleotide polymorphisms (SNPs) significantly associated with these markers as IVs. The outcome variable of interest was DVT. To estimate the causal effects between muscle strength and DVT, we performed a 2-sample MR analysis (Fig. 1).

### 2.2. Genetic associations with exposures and outcomes

The genome-wide association study (GWAS) summary data for hand-grip strength were obtained from a meta-analysis involving 22 cohorts and a total of 256,523 individuals of European descent who were aged 60 years or older.^[20]^ Hand-grip strength was assessed based on the European Working Group on Sarcopenia in Older People’s (EWGSOP) definition, which set the thresholds at 30 kg for men and 20 kg for women.^[21]^ According to this definition, 46,596 participants (18.9% of the total sample) reported muscular weakness. Additionally, the Foundations of the National Institutes of Health (FNIH) definition was used, which set the thresholds at 26 kg for men and 16 kg for women. Under this definition, 19,345 participants (7.6% of the total sample) reported muscular weakness.^[22]^ The data utilized in our study was obtained from the FinnGen study.^[23]^ Specifically, we utilized the R6 release data, which included corresponding genome-wide association analyses involving 5632 DVT cases and up to 254,771 controls. In the FinnGen study, the association tests were adjusted for age, sex, the first 10 genetic principal components, and genotyping batch. All studies included in GWASs have received approval from the respective review committees; hence, ethical approval and informed consent are deemed unnecessary.

### 2.3. Selection of instrumental variables

In our study, we implemented a stringent selection process to identify SNPs for our analysis, focusing on their linkage disequilibrium (LD) patterns. Initially, we identified SNPs associated with muscle strength at genome-wide significance (*P* < 5e^−7^). To ensure independence among the SNPs, we retained only those pairs with an LD (*r*²) value exceeding 0.001 within a 10,000 kb range and a minor allele frequency (MAF) above 5%. These thresholds are commonly applied in MR studies to minimize the risk of bias from correlated variants and ensure sufficient power for detecting associations. Similar approaches have been used in previous studies.^[24,25]^ Through this process, we obtained independent SNPs that exhibited the strongest associations with the exposure variable. Next, we examined the overlap between the remaining SNPs and the relevant GWAS database to establish a significant correlation with DVT. This step verified the suitability of these SNPs as IVs. To address potential weak instrumental bias resulting from sample overlap, our primary analyses were conducted using subjects from the FinnGen research project. To quantify the percentage of variability in muscle mass explained by each SNP, we obtained the *R*^2^ value through the MR Steiger directionality test. This test considered the number of samples (N) from the exposure GWAS and the number of SNPs (*K*) in the IVs. Specifically, when assessing a single SNP, *K* is equal to 1. The *R*^2^ value was calculated using Equation 1: *R*^2^ = 2 × EAF × (1 − EAF)×β^2^. We utilized the *F* statistic to evaluate the presence of weak IVs bias, with a value below 10 indicating a weak IV bias.^[26]^ The *F* statistic was computed using Equation 2: *F* = N − *K* − 1/*K* × *R*^2^/(1 − *R*^2^).

### 2.4. Statistical analysis

To investigate the potential causal relationship between genetic risk of exposure and outcome, we employed several statistical methods to address pleiotropy bias, including inverse-variance weighting (IVW), MR-Egger, and weighted median (WM) approaches. The IVW method utilizes a meta-analysis framework to combine Wald estimates from individual single nucleotide SNPs and obtain an overall estimate of the effect of muscle strength on DVT. In cases where significant heterogeneity (*P* < .05) was observed, we employed the random effects IVW model.^[27]^ To account for potential pleiotropy, the MR-Egger method was utilized. This method incorporated an intercept term into the IVW analysis to assess the average pleiotropic effect of IVs. However, it is important to note that the accurate estimation of the MR-Egger method can be influenced by external genetic variation, potentially limiting its accuracy.^[28]^ In addition, we employed the WM method as a supplementary approach to provide a robust and consistent estimate of the causal effect. This method is advantageous as it can produce reliable estimates even when approximately 50% of the genetic variants function as invalid instruments.^[29]^ The weighted mode method can only evaluate the causal validity according to the cluster with the largest number of SNPs, but cannot estimate the bandwidth parameter.^[30]^

### 2.5. Sensitivity analysis

To ensure the robustness and reliability of our MR causal impact estimate, we conducted sensitivity analyses.^[31]^ Initially, we employed the MR-Egger approach to assess the presence of horizontal pleiotropy among the selected SNPs.^[32]^ If the *P* value of the intercept term was <.05, it indicated the potential presence of pleiotropic effects of IVs. Conversely, if the *P* value of the intercept term was not <.05, there was no evidence of horizontal pleiotropy across the chosen IVs.

Next, we utilized the MR Pleiotropy Residual Sum and Outlier (MR-PRESSO) test to identify outliers and obtain a robust estimate after outlier correction. A sensitivity analysis was performed to evaluate substantial distortions in the IVW causal estimate before and after applying the MR-PRESSO adjustment. This involved replicating the random-effects IVW analysis by iteratively removing individual SNPs and comparing the overall results. The sensitivity of each SNP was determined based on the extent of changes observed in the findings before and after its removal.

For reporting MR findings, odds ratios (ORs) with 95% confidence intervals (CIs) per standard deviation were used for dichotomous variables, while beta values (β) with 95% CIs per standard deviation were employed for continuous variables. Consistent reporting of estimates was maintained throughout the study. A 2-tailed *P* value threshold of .05 was considered statistically significant. The statistical analysis was performed using the 2-sample MR and MR-PRESSO packages in R version 4.1.1.^[33]^

## 3. Results

The information for IVs was found in Tables S1, Supplemental Digital Content, http://links.lww.com/MD/N861 and S2, Supplemental Digital Content, http://links.lww.com/MD/N861. We obtained 21 and 14 LD-independent (*r*^2^ < 0.001) IVs that achieved genome-wide significance levels for hand grip strength (EWGSOP) and hand grip strength (FNIH). IVW in a random-effects model was used according to the results of the heterogeneity test. IVW analysis showed EWGSOP (OR = 0.702, 95% CI 0.511–0.963, *P* = .029) and FNIH (OR = 0.715, 95% CI 0.570–0.898, *P* = .004) were causally and negatively associated with DVT (Table 1). The MR-Egger and MR-PRESSO analysis did not detect any directional pleiotropy for our selected IVs (Table 2).

**Table 1:** MR estimates of the causal association between muscle strength and DVT.

Exposure	SNPs	IVW	WM	MR-Egger
		OR (95% CI)	OR (95% CI)	OR (95% CI)
HGS (EWGSOP)	21	0.702 (0.511–0.964)	0.792 (0.564–1.112)	0.484 (0.154–1.524)
	*P* value	**.029** *	.178	.230
HGS (FNIH)	14	0.715 (0.570–0.898)	0.595 (0.309–1.146)	0.685 (0.512–0.917)
	*P* value	**.004** *	.147	**.011** *

HGS = hand grip strength, IVW = inverse-variance weighting, MR = Mendelian randomization, WM = weighted median.

* Statistically significant difference.

**Table 2:** Sensitivity analysis of the causal association between hand grip strength and DVT.

Exposure	Cochran *Q*		MR-Egger		MR-PRESSO global test
	*Q* value	*P*	Intercept	*P*	*P*
HGS (EWGSOP)	41.155	.004	0.020	.516	.162
HGS (FNIH)	13.924	.352	0.017	.569	.371

HGS = hand grip strength.

**Figure 2.**
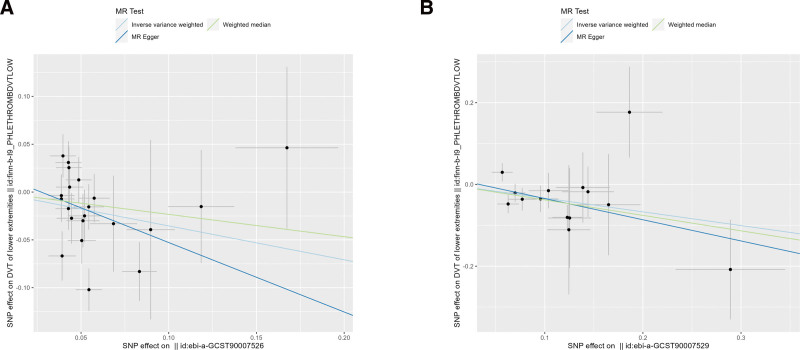
Scatter plots of causality. The slope of each line corresponds to the estimated MR effect in different models. (A) Hand grip strength (EWGSOP; ebi-a-GCST90007526); (B) hand grip strength (FNIH; ebi-a-GCST90007529). MR = Mendelian randomization.

**Figure 3.**
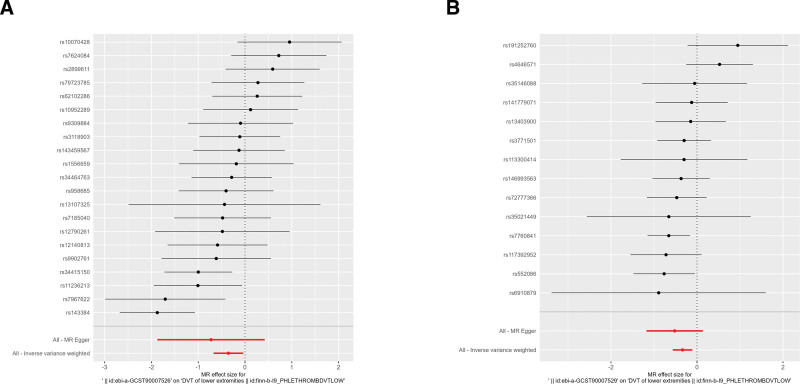
Forest plot for individual causal effect estimate. (A) Hand grip strength (EWGSOP; ebi-a-GCST90007526); (B) Hand grip strength (FNIH; ebi-a-GCST90007529).

The scatter and forest plots of the causal relationships between hand grip strength and the risk of DVT were shown in Figures 2 and 3. The funnel plot (Fig. S1, Supplemental Digital Content, http://links.lww.com/MD/N862) shows that when a single SNP is used as an IV, the points representing causal effects are symmetrically distributed, which indicates that causal effects are less likely to be affected by potential bias. In the leave-one-out analysis (Fig. S2, Supplemental Digital Content, http://links.lww.com/MD/N862), the removal of each SNP in turn has little effect on the results, indicating that there is no single SNP which has a significant influence on the overall causal effect estimation. The details of the sensitivity analysis are presented in Table 2.

## 4. Discussion

Our MR analysis provided compelling evidence supporting a causal negative association between hand grip strength and the risk of DVT. For low hand grip strength (EWGSOP), the OR was 0.702, indicating a 29.8% lower risk of DVT in individuals with stronger hand grip strength. Similarly, for low hand grip strength (FNIH), the OR was 0.715, suggesting a 28.5% lower risk of DVT in individuals with stronger hand grip strength. The ORs indicated a clear inverse relationship, suggesting that a higher hand grip strength is associated with a lower risk of developing DVT. The MR-Egger analysis did not detect any directional pleiotropy, suggesting that our results were unlikely to be influenced by confounding factors.

These findings provide robust evidence supporting the notion that hand grip strength plays a significant role in influencing the risk of DVT. It has been well-documented that muscle strength is integral to overall health and venous function, with studies like those on respiratory muscle training and injury prevention highlighting broader roles for muscle strength.^[34]^ While these studies focus on different health outcomes, the underlying concept of muscle strength’s importance aligns with our findings on DVT. One possible mechanism is related to the role of muscle strength in maintaining venous function and blood flow.^[35]^ Adequate muscle strength, including hand grip strength, contributes to the proper functioning of the muscle-vein pump system, which assists in venous return and prevents blood stasis.^[36]^ Weaker muscle strength may lead to impaired muscle-vein pump function, resulting in reduced blood flow and increased susceptibility to DVT formation.^[37,38]^ Supporting this mechanism, a population-based study conducted on Olmsted County residents with no prior history of venous thromboembolism found that reduced calf pump function, as measured by venous plethysmography, was associated with an increased risk of venous thromboembolism, particularly lower extremity proximal DVT.^[12]^ This finding suggests that compromised muscle-vein pump function may contribute to the development of DVT.^[39]^

Furthermore, muscle strength and DVT risk may also be influenced by systemic inflammation and coagulation factors, both closely related to physical fitness. Chronic inflammation, often linked to decreased muscle mass and strength, has been identified as a key factor in promoting a prothrombotic state, increasing the risk of venous thrombosis.^[40,41]^ Regular muscle contractions during exercise stimulate the release of various molecules and growth factors that promote healthy endothelial function.^[42,43]^ The endothelium, the inner lining of blood vessels, helps regulate blood clotting and vasodilation.^[44–46]^ This improved endothelial function, combined with evidence from animal studies demonstrating the potential role of exercise and muscle strength in promoting thrombus resolution and recanalization, highlights the benefits of exercise in DVT management and prevention.^[14]^ By improving muscle strength, maintaining a balanced coagulation system may contribute to a reduced risk of thrombosis. Many recent clinical studies have specifically reported that exercise is sufficiently safe for DVT, as it does not increase the incidence of pulmonary embolism.^[47–49]^ Muscle tissue is metabolically active and plays a crucial role in glucose and lipid metabolism.^[50]^ Conditions like obesity and metabolic syndrome, characterized by decreased muscle mass and strength, are associated with an increased risk of DVT.^[10,51]^ It is plausible that maintaining or increasing muscle mass through regular exercise and resistance training may positively influence metabolic parameters, reducing the risk of metabolic abnormalities that contribute to DVT.^[52]^ Given the multifactorial nature of DVT, future research could also investigate how other physiological factors such as endothelial dysfunction, insulin resistance, and lipid metabolism interact with muscle strength to influence DVT risk. These interactions may further clarify the complex pathways involved in venous thrombosis, providing opportunities for targeted prevention strategies that address both muscle strength and metabolic health.^[53,54]^ There is no doubt that further clinical studies or experimental validations are needed to confirm that resistance training is helpful in reducing the occurrence of DVT, which is also the direction of our further research.

It is important to acknowledge certain limitations of our study. First, the use of hand grip strength as a proxy for overall muscle strength may not capture the full complexity of muscle health. Future research should explore other measures of muscle strength and evaluate their associations with DVT risk. Second, while our MR analysis helps establish causality, it is essential to consider the context-specific factors that influence the relationship between muscle strength and DVT, such as age, comorbidities, and lifestyle factors. While we were able to assess hand grip strength as a proxy for overall muscle strength, we acknowledge that DVT primarily affects the lower extremities. Evaluating the muscle strength of the lower limbs, particularly in key muscle groups involved in venous function, would provide a more direct assessment of the association between muscle strength and DVT risk. Third, the GWAS data used in the study were all from European origin, so whether the findings can be replicated in other samples of other ethnicities is not known. Forth, due to the limitations of GWAS data, there was no stratified analysis by gender, so larger and more detailed GWAS data will be needed for future studies that will lead to more comprehensive and reliable conclusions.

## 5. Conclusion

Our study provides strong evidence that low hand grip strength is causally associated with a decreased risk of DVT. These findings highlight the importance of muscle strength in reducing the likelihood of developing this potentially serious vascular condition. Future research, including randomized controlled trials, should be conducted to explore the underlying mechanisms and evaluate the effectiveness of interventions targeting muscle strength to prevent DVT and its associated complications.

## Author contributions

**Conceptualization:** Bin Pu.

**Formal analysis:** Ziwei Jiang.

**Funding acquisition:** Bin Pu.

**Investigation:** Daoze Ke, Changfan Li.

**Methodology:** Yijia Gao, Hui Chen, Changfan Li.

**Project administration:** Yijia Gao.

**Resources:** Hui Chen, Daoze Ke, Ziwei Jiang.

**Software:** Daoze Ke, Ziwei Jiang.

**Supervision:** Hui Chen.

**Validation:** Daoze Ke, Bin Pu.

**Visualization:** Daoze Ke.

**Writing – original draft:** Yijia Gao, Bin Pu.

**Writing – review & editing:** Yijia Gao, Hui Chen.

## Supplementary Material



## References

[1] Memtsoudis SG Della Valle AG Besculides MC Esposito M Koulouvaris P Salvati EA. Risk factors for perioperative mortality after lower extremity arthroplasty: a population-based study of 6,901,324 patient discharges. J Arthroplasty. 2010;25:19–26. 19106028 10.1016/j.arth.2008.11.010

[2] Lee JS Moon T Kim TH. Deep vein thrombosis in patients with pulmonary embolism: prevalance, clinical significance and outcome. Vasc Specialist Int. 2016;32:166–74. 28042556 10.5758/vsi.2016.32.4.166 PMC5198763

[3] Kesieme E Kesieme C Jebbin N Irekpita E Dongo A. Deep vein thrombosis: a clinical review. J Blood Med. 2011;2:59–69. 22287864 10.2147/JBM.S19009 PMC3262341

[4] Naess IA Christiansen SC Romundstad P Cannegieter SC Rosendaal FR Hammerstrøm J. Incidence and mortality of venous thrombosis: a population-based study. J Thromb Haemost. 2007;5:692–9. 17367492 10.1111/j.1538-7836.2007.02450.x

[5] Pastori D Cormaci VM Marucci S. A comprehensive review of risk factors for venous thromboembolism: from epidemiology to pathophysiology. Int J Mol Sci . 2023;24:3169. 36834580 10.3390/ijms24043169 PMC9964264

[6] Wadajkar AS Santimano S Rahimi M Yuan B Banerjee S Nguyen KT. Deep vein thrombosis: current status and nanotechnology advances. Biotechnol Adv. 2013;31:504–13. 22940402 10.1016/j.biotechadv.2012.08.004 PMC5111858

[7] Cushman M. Epidemiology and risk factors for venous thrombosis. Semin Hematol. 2007;44:62–9. 17433897 10.1053/j.seminhematol.2007.02.004 PMC2020806

[8] Vigh-Larsen JF Junge N Cialdella-Kam L. Testing in intermittent sports-importance for training and performance optimization in adult athletes. Med Sci Sports Exerc. 2024;56:1505–37. 39004796 10.1249/MSS.0000000000003442

[9] Bond CW Hackney KJ Brown SL Noonan BC. Blood flow restriction resistance exercise as a rehabilitation modality following orthopaedic surgery: a review of venous thromboembolism risk. J Orthop Sports Phys Ther. 2019;49:17–27. 30208794 10.2519/jospt.2019.8375

[10] Di Nisio M Di Iorio A Porreca E. Obesity, poor muscle strength, and venous thromboembolism in older persons: the InCHIANTI study. J Gerontol A Biol Sci Med Sci. 2011;66:320–5. 21112964 10.1093/gerona/glq207 PMC3041471

[11] Shin JM Hong SJ Choi KH. Low relative muscle volume: correlation with prevalence of venous thromboembolism following total knee arthroplasty. PLoS One. 2019;14:e0210800. 30835735 10.1371/journal.pone.0210800 PMC6400339

[12] Houghton DE Ashrani A Liedl D. Reduced calf muscle pump function is a risk factor for venous thromboembolism: a population-based cohort study. Blood. 2021;137:3284–90. 33657212 10.1182/blood.2020010231 PMC8351902

[13] Leknessund OGR Morelli VM Strand BH Hansen J-B Brækkan SK. Hand grip strength and risk of incident venous thromboembolism: the Tromso study. Res Pract Thromb Haemost. 2022;6:e12833. 36349264 10.1002/rth2.12833 PMC9634490

[14] Wu C Li X Zhao H. Resistance exercise promotes the resolution and recanalization of deep venous thrombosis in a mouse model via SIRT1 upregulation. BMC Cardiovasc Disord. 2023;23:18. 36639616 10.1186/s12872-022-02908-y PMC9837998

[15] Rosendaal FR. Venous thrombosis: a multicausal disease. Lancet. 1999;353:1167–73. 10209995 10.1016/s0140-6736(98)10266-0

[16] Kahn SR Shrier I Kearon C. Physical activity in patients with deep venous thrombosis: a systematic review. Thromb Res. 2008;122:763–73. 18078981 10.1016/j.thromres.2007.10.011

[17] Padberg FT Jr Johnston MV Sisto SA. Structured exercise improves calf muscle pump function in chronic venous insufficiency: a randomized trial. J Vasc Surg. 2004;39:79–87. 14718821 10.1016/j.jvs.2003.09.036

[18] Davies NM Holmes MV Davey Smith G. Reading Mendelian randomisation studies: a guide, glossary, and checklist for clinicians. BMJ. 2018;362:k601. 30002074 10.1136/bmj.k601 PMC6041728

[19] Lawlor DA Harbord RM Sterne JA Timpson N Davey Smith G. Mendelian randomization: using genes as instruments for making causal inferences in epidemiology. Stat Med. 2008;27:1133–63. 17886233 10.1002/sim.3034

[20] Jones G Trajanoska K Santanasto AJ. Genome-wide meta-analysis of muscle weakness identifies 15 susceptibility loci in older men and women. Nat Commun. 2021;12:654. 33510174 10.1038/s41467-021-20918-w PMC7844411

[21] Cruz-Jentoft AJ Baeyens JP Bauer JM; European Working Group on Sarcopenia in Older People. Sarcopenia: European consensus on definition and diagnosis: report of the European Working Group on Sarcopenia in older people. Age Ageing. 2010;39:412–23. 20392703 10.1093/ageing/afq034 PMC2886201

[22] Alley DE Shardell MD Peters KW. Grip strength cutpoints for the identification of clinically relevant weakness. J Gerontol A Biol Sci Med Sci. 2014;69:559–66. 24737558 10.1093/gerona/glu011 PMC3991145

[23] Kurki MI Karjalainen J Palta P; FinnGen. Author correction: FinnGen provides genetic insights from a well-phenotyped isolated population. Nature. 2023;615:E19. 36829046 10.1038/s41586-023-05837-8 PMC10017492

[24] Hebbar P Abubaker JA Abu-Farha M. Genome-wide landscape establishes novel association signals for metabolic traits in the Arab population. Hum Genet. 2021;140:505–28. 32902719 10.1007/s00439-020-02222-7 PMC7889551

[25] Zhou K Zhang Q Yuan Z Yan Y Zhao Q Wang J. Plasma fatty acids and attention deficit hyperactivity disorder: a Mendelian randomization investigation. Front Psychiatry. 2024;15:1368942. 38764473 10.3389/fpsyt.2024.1368942 PMC11099612

[26] Staiger DO Stock JH. Instrumental Variables Regression with Weak Instruments. Cambridge, Mass., USA: National Bureau of Economic Research; 1994.

[27] Bowden J Del Greco MF Minelli C. A framework for the investigation of pleiotropy in two-sample summary data Mendelian randomization. Stat Med. 2017;36:1783–802. 28114746 10.1002/sim.7221 PMC5434863

[28] Zheng J Frysz M Kemp JP Evans DM Davey Smith G Tobias JH. Use of Mendelian randomization to examine causal inference in osteoporosis. Front Endocrinol (Lausanne). 2019;10:807. 31824424 10.3389/fendo.2019.00807 PMC6882110

[29] Bowden J Davey Smith G Haycock PC Burgess S. Consistent estimation in Mendelian randomization with some invalid instruments using a weighted median estimator. Genet Epidemiol. 2016;40:304–14. 27061298 10.1002/gepi.21965 PMC4849733

[30] Zhou J Ye Z Wei P. Effect of basal metabolic rate on osteoporosis: a Mendelian randomization study. Front Public Health. 2023;11:1096519. 36817914 10.3389/fpubh.2023.1096519 PMC9929187

[31] Burgess S Bowden J Fall T Ingelsson E Thompson SG. Sensitivity analyses for robust causal inference from Mendelian randomization analyses with multiple genetic variants. Epidemiology. 2017;28:30–42. 27749700 10.1097/EDE.0000000000000559 PMC5133381

[32] Bowden J Davey Smith G Burgess S. Mendelian randomization with invalid instruments: effect estimation and bias detection through Egger regression. Int J Epidemiol. 2015;44:512–25. 26050253 10.1093/ije/dyv080 PMC4469799

[33] Yavorska OO Burgess S. MendelianRandomization: an R package for performing Mendelian randomization analyses using summarized data. Int J Epidemiol. 2017;46:1734–9. 28398548 10.1093/ije/dyx034 PMC5510723

[34] Xu D Zhou H Quan W. New insights optimize landing strategies to reduce lower limb injury risk. Cyborg Bionic Syst. 2024;5:0126. 38778877 10.34133/cbsystems.0126 PMC11109754

[35] Lopes KG Farinatti P Bottino DA. Exercise with blood flow restriction improves muscle strength and mass while preserving the vascular and microvascular function and structure of older adults. Clin Hemorheol Microcirc. 2022;82:13–26. 35599474 10.3233/CH-221395

[36] Ichinose M Ichinose-Kuwahara T Kondo N Nishiyasu T. Increasing blood flow to exercising muscle attenuates systemic cardiovascular responses during dynamic exercise in humans. Am J Physiol Regul Integr Comp Physiol. 2015;309:R1234–42. 26377556 10.1152/ajpregu.00063.2015 PMC4666933

[37] Lv B Wang H Li W. Admission prevalence and risk factors of deep vein thrombosis in patients with spinal cord injury complicated with cervical fractures. Clin Appl Thromb Hemost. 2022;28:10760296221108969. 35763449 10.1177/10760296221108969 PMC9247371

[38] Giorgi Pierfranceschi M Donadini MP Dentali F. The short- and long-term risk of venous thromboembolism in patients with acute spinal cord injury: a prospective cohort study. Thromb Haemost. 2013;109:34–8. 23223906 10.1160/TH12-06-0390

[39] Akesson H Brudin L Dahlstrom JA Eklöf B Ohlin P Plate G. Venous function assessed during a 5 year period after acute ilio-femoral venous thrombosis treated with anticoagulation. Eur J Vasc Surg. 1990;4:43–8. 2323420 10.1016/s0950-821x(05)80037-4

[40] Grimnes G Isaksen T Tichelaar Y. C-reactive protein and risk of venous thromboembolism: results from a population-based case-crossover study. Haematologica. 2018;103:1245–50. 29674505 10.3324/haematol.2017.186957 PMC6029539

[41] Montilla-Garcia A Tejada MA Perazzoli G. Grip strength in mice with joint inflammation: a rheumatology function test sensitive to pain and analgesia. Neuropharmacology. 2017;125:231–42. 28760650 10.1016/j.neuropharm.2017.07.029

[42] Green DJ Maiorana A O’Driscoll G Taylor R. Effect of exercise training on endothelium-derived nitric oxide function in humans. J Physiol. 2004;561:1–25. 15375191 10.1113/jphysiol.2004.068197 PMC1665322

[43] Di Francescomarino S Sciartilli A Di Valerio V Di Baldassarre A Gallina S. The effect of physical exercise on endothelial function. Sports Med. 2009;39:797–812. 19757859 10.2165/11317750-000000000-00000

[44] Sandoo A van Zanten JJ Metsios GS Carroll D Kitas GD. The endothelium and its role in regulating vascular tone. Open Cardiovasc Med J. 2010;4:302–12. 21339899 10.2174/1874192401004010302 PMC3040999

[45] van Hinsbergh VW. Endothelium--role in regulation of coagulation and inflammation. Semin Immunopathol. 2012;34:93–106. 21845431 10.1007/s00281-011-0285-5 PMC3233666

[46] Rajendran P Rengarajan T Thangavel J. The vascular endothelium and human diseases. Int J Biol Sci. 2013;9:1057–69. 24250251 10.7150/ijbs.7502 PMC3831119

[47] Aschwanden M Labs KH Engel H. Acute deep vein thrombosis: early mobilization does not increase the frequency of pulmonary embolism. Thromb Haemost. 2001;85:42–6. 11204585

[48] Isma N Johanssson E Bjork A. Does supervised exercise after deep venous thrombosis improve recanalization of occluded vein segments? A randomized study. J Thromb Thrombolysis. 2007;23:25–30. 17186396 10.1007/s11239-006-9010-y

[49] Gong JM Du JS Han DM. Implications of bed rest for patients with acute deep vein thrombosis: a qualitative study. Patient Prefer Adherence. 2020;14:1659–67. 32982190 10.2147/PPA.S271481 PMC7509328

[50] Morales PE Bucarey JL Espinosa A. Muscle lipid metabolism: role of lipid droplets and perilipins. J Diabetes Res. 2017;2017:1789395. 28676863 10.1155/2017/1789395 PMC5476901

[51] Stewart LK Kline JA. Metabolic syndrome increases risk of venous thromboembolism recurrence after acute deep vein thrombosis. Blood Adv. 2020;4:127–35. 31917844 10.1182/bloodadvances.2019000561 PMC6960475

[52] Nascimento DDC Rolnick N Neto IVS Severin R Beal FLR. A useful blood flow restriction training risk stratification for exercise and rehabilitation. Front Physiol. 2022;13:808622. 35360229 10.3389/fphys.2022.808622 PMC8963452

[53] Park SH Kim DJ Plank LD. Association of grip strength with non-alcoholic fatty liver disease: investigation of the roles of insulin resistance and inflammation as mediators. Eur J Clin Nutr. 2020;74:1401–9. 32152511 10.1038/s41430-020-0591-x

[54] Vrints CJM. Deep venous thrombosis and endothelial dysfunction in cancer: prevention and early initiated rehabilitation should be integral to a cardio-oncology programme. Eur J Prev Cardiol. 2022;29:1244–7. 34463767 10.1093/eurjpc/zwab117

