# Supplementary material for: Effect of muscle strength on deep vein thrombosis: A Mendelian randomization study
Source: Medicine (Baltimore). 2024 Nov 8;103(45):e40138. doi: 10.1097/MD.0000000000040138 (PMC11557092; doi:10.1097/MD.0000000000040138)
Supplement: Supplementary file 2 [file medi-103-e40138-s002.docx]

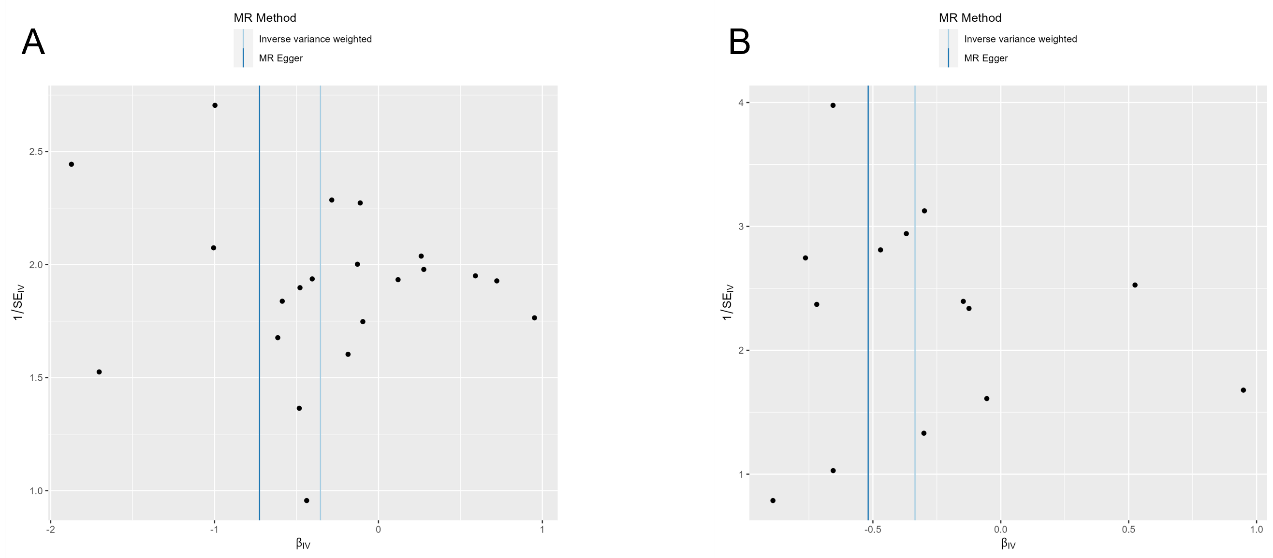


Supplementary Figure 1. Funnel plot of causality. (A) Hand grip strength (EWGSOP; ebi-a-GCST90007526); (B) Hand grip strength (FNIH; ebi-a-GCST90007529).


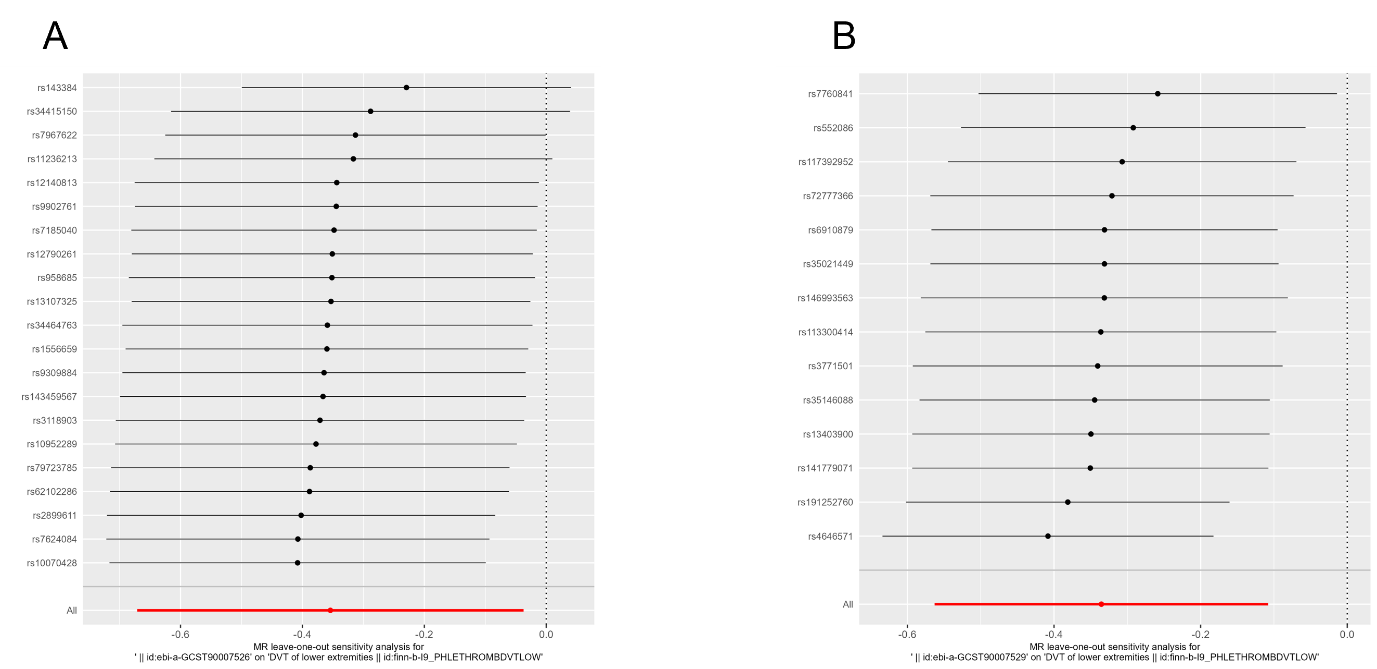
Supplementary Figure 2. Forest plot for leave-one-out analysis, with each point denoting the causal effect by IVW after removing the specific SNP. (A) Hand grip strength (EWGSOP; ebi-a-GCST90007526); (B) Hand grip strength (FNIH; ebi-a-GCST90007529).
